# Live cell imaging of genomic loci using dCas9-SunTag system and a bright fluorescent protein

**DOI:** 10.1007/s13238-017-0460-0

**Published:** 2017-08-21

**Authors:** Huiying Ye, Zhili Rong, Ying Lin

**Affiliations:** Cancer Research Institute, School of Basic Medical Sciences, Southern Medical University, Guangzhou, 510515 China

**Dear Editor,**

CRISPR-Cas9 (clustered regularly interspaced short palindromic repeats-CRISPR associated) systems have been harnessed for kinds of genome manipulation, including gene editing, transcription regulation, and chromosome loci imaging (Dominguez et al., 2016; Komor et al., 2017). A typical engineered CRISPR-Cas9 system is composed of a Cas9 protein and a single guide RNA (sgRNA), which could form a protein/RNA complex to recognize and cleave DNA sequence (Hsu et al., 2014; Wright et al., 2016). Based on nuclease-deactivated Cas9, termed dCas9, the CRISPR-Cas9 system can be used as an imaging tool to label genomic loci and visualize dynamic changes of chromosomes (Chen et al., 2013; Cheng et al., 2016; Fu et al., 2016; Ma et al., 2016; Qin et al., 2017; Wang et al., 2016). An EGFP-tagged dCas9 bound with a structurally optimized sgRNA enables visualization of repetitive genomic sequences in live cells (Chen et al., 2013). A CRISPRainbow system could simultaneously image up to six genomic loci in a single cell (Ma et al., 2016). However, these approaches have an obvious disadvantage, the fluorescent signal is relative weak and the signal-to-noise ratio is low, and thus the application to low or no repeats DNA sequences is limited.

SunTag system is a protein-tagging system for signal amplification, consisting of an array of repeating peptide and an antibody-fusion protein, which can bind to each other (Tanenbaum et al., 2014). mNeonGreen, a monomeric yellow-green fluorescent protein derived from a tetrameric fluorescent protein from the cephalochordate *Branchiostoma lanceolatum*, is reported to be the brightest monomeric green or yellow fluorescent protein so far described (Shaner et al., 2013). In comparison experiments, mNeonGreen showed excellent properties that were superior to the most commonly used green and yellow fluorescent proteins, and thus held great potential for imaging.

Considering their unique properties, we expected that application of SunTag system and mNeonGreen to CRISPR/Cas9-based imaging might increase signal intensity, as well as signal-to-noise ratio. Therefore, 24 copies of GCN4 peptide were fused to the C-terminus of dCas9 (dCas9-SunTag), and GCN4 peptide binding single-chain variable fragment antibody (scFv-GCN4) was fused to superfolder-GFP (sfGFP), mNeonGreen, or three-tandem-repeats of mNeonGreen (3XmNeonGreen). As sgRNA structure could significantly affect imaging quality, a sgRNA scaffold optimized by A-U base pair flip and hairpin extension was used to eliminate protein aggregation and increase bright dot number (Chen et al., 2013). The schematic of the imaging strategy was shown in Fig. 1A. To test the hypothesis, HEK293T cells were co-transfected with dCas9-SunTag, a telomere-targeted sgRNA, and sfGFP, mNeonGreen, or 3XmNeonGreen, respectively. As shown in Fig. 1B and Movies S1, S2 and S3, all the fluorescent proteins formed puncta in cell nuclei. In a quantitative assay, about 26% cells contained 61–80 foci and about 5% cells contained 81–100 foci in sfGFP transfected cells, and about 27% cells contained 61–80 foci and about 24% cells contained 81–100 foci in mNeonGreen transfected cells, while about 38% cells contained 61–80 foci and about 10% cells contained 81–100 foci in 3XmNeonGreen transfected cells. Since the expected telomere number is about 92 at G_1_ cell cycle stage in a diploid human cell, the mNeonGreen strategy is the optimal one to visualize most telomere loci in a cell, compared with the other two strategies (Fig. 1C). To further assess the specificity and efficiency of telomere labeling by the three strategies, fluorescence *in situ* hybridization (FISH) assay using a Cy5-tagged telomere-specific-probe and immunofluorescence staining assay using a primary antibody against HA tag (each fluorescence protein contains a HA tag) and an Alexa488-labeled secondary antibody were performed in the same cells (Schmitt et al., 2010). The results showed that FISH and sfGFP, mNeonGreen, and 3XmNeonGreen signals were perfectly matched, indicating that the foci of sfGFP, mNeonGreen, and 3XmNeonGreen were indeed corresponding to telomeres and that the efficiency is similar between CRISPR imaging and FISH assay (Fig. 1D). As shown in Figure 1B and 1D, the fluorescence signal intensity and signal-to-noise ratio were obviously different, and thus a quantitative assay was performed. The fluorescence intensity of 3XmNeonGreen was higher than the other two (*P* < 0.001), while there was no statistical significant difference between sfGFP and mNeonGreen (Fig. 1E). And the foci in cells expressing mNeonGreen showed higher signal-to-noise ratio than that in cells expressing the other two fluorescent proteins (Fig. 1F). To interrogate intranuclear dynamics of telomeres in living cells, time-series about the movement of telomeres were acquired simultaneously by time-lapse microscopy. Single-particle tracking with different fluorescent proteins revealed that the telomere movement is confined diffusion (Fig. S1A and Movies S4, S5 and S6). To test whether the CRISPR approaches could be applied to image low-repeat chromosome loci, the program Tandem Repeat Finder was employed to find repeat sequences in human genome (Benson, 1999), and a locus containing 21 copies of exact repeats on chromosome 5 (Ch5R), and a locus containing 15 copies of exact repeats on chromosome 14 (Ch14R) were successfully visualized with any of the three strategies.

**Figure 1:**
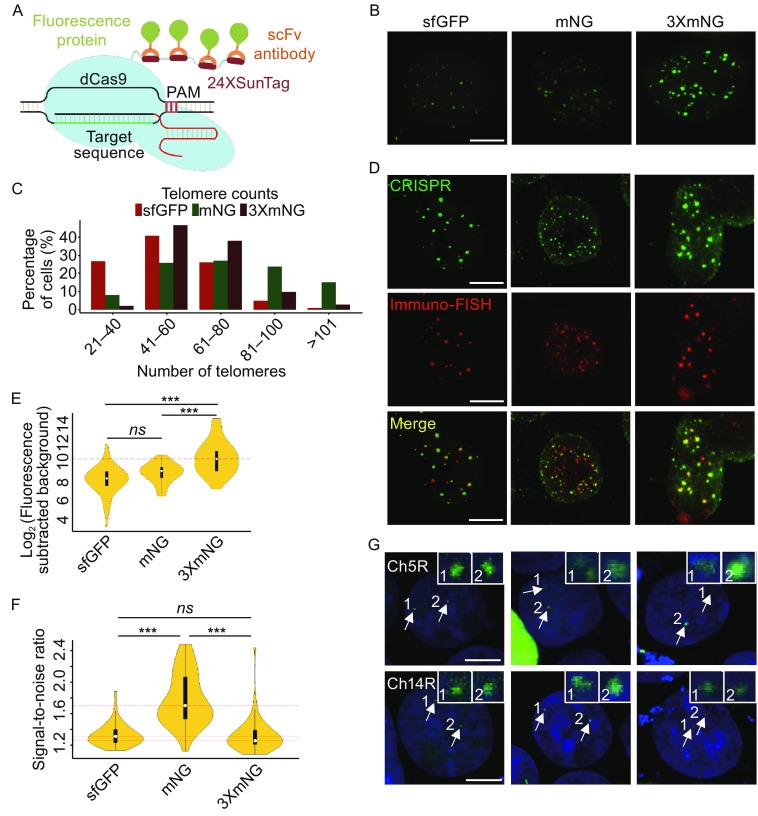
**Live cell imaging of genomic loci using dCas9-SunTag system and a bright fluorescent protein**. (A) Schematic of dCas9-SunTag system combined with sfGFP, mNeonGreen (mNG) or 3XmNeonGreen (3XmNG). (B) Visualizing human telomeres with CRISPR/Cas9 system using different fluorescent proteins. HEK293T cells were transfected with sgTelomere and dCas9-SunTag system combined with sfGFP, mNeonGreen and 3XmNeonGreen, respectively. 48 h after transfection, cells were imaged by confocal laser scanning microscope. Scale bar, 2 μm. (C) Histogram of telomere punta counts per cell detected by sfGFP, mNeonGreen, and 3XmNeonGreen (N = 66, 62, and 60 cells, respectively). (D) Co-localization of telomeres using sfGFP, mNeonGreen, 3XmNeonGreen (top), and FISH (middle). Scale bar, 5 μm. (E and F) CRISPR fluorescence intensity and signal-to-noise ratio of labelled telomere foci. N = 50 cells for each analysis and the dot within the vioplot represents the median value. (G) Two low-repeat genomic loci were labeled with different fluorescence proteins. Ch5R and Ch14R sites contain 21 and 15 copies of repeats, respectively. The experiment procedure is similar to telomere labeling. Nuclei were counterstained with the live staining dye Hoechst33342. Scale bar, 5 μm. All images are achieved by maximum intensity projections from Z-stacks

In this study, we combined CRISPR/Cas9 technology, SunTag system, and fluorescent proteins to image high-repetitive and low-repetitive chromosome loci in human cells. mNeonGreen labeling resulted in the best signal-to-noise ratio and 3XmNeonGreen labeling leaded to the strongest signal intensity among sfGFP, mNeonGreen, and 3XmNeonGreen imaging strategies. In addition, all the three approaches could be used to image low-repetitive loci, as less to15 copies of repeats. In summary, we developed novel tools to visualize chromosome loci in live cells with variant signal-to-noise ratios and signal intensities, and therefore broaden the adaptability of CRISPR/Cas9 system based imaging methods.

## Electronic supplementary material

Below is the link to the electronic supplementary material.

Supplementary material 1 (AVI 351 kb)

Supplementary material 2 (AVI 471 kb)

Supplementary material 3 (AVI 661 kb)

Supplementary material 4 (AVI 94 kb)

Supplementary material 5 (AVI 293 kb)

Supplementary material 6 (AVI 97 kb)

Supplementary material 7 (PDF 270 kb)

## References

[CR1] Benson G (1999). Tandem repeats finder: a program to analyze DNA sequences. Nucleic Acids Res.

[CR2] Chen B, Gilbert LA, Cimini BA, Schnitzbauer J, Zhang W (2013). Dynamic imaging of genomic loci in living human cells by an optimized CRISPR/Cas system. Cell.

[CR3] Cheng AW, Jillette N, Lee P, Plaskon D, Fujiwara Y (2016). Casilio: a versatile CRISPR-Cas9-Pumilio hybrid for gene regulation and genomic labeling. Cell Res.

[CR4] Dominguez AA, Lim WA, Qi LS (2016). Beyond editing: repurposing CRISPR-Cas9 for precision genome regulation and interrogation. Nat Rev Mol Cell Biol.

[CR5] Fu Y, Rocha PP, Luo VM, Raviram R, Deng Y (2016). CRISPR-dCas9 and sgRNA scaffolds enable dual-colour live imaging of satellite sequences and repeat-enriched individual loci. Nat Commun.

[CR6] Hsu PD, Lander ES, Zhang F (2014). Development and applications of CRISPR-Cas9 for genome engineering. Cell.

[CR7] Komor AC, Badran AH, Liu DR (2017). CRISPR-based technologies for the manipulation of eukaryotic genomes. Cell.

[CR8] Ma H, Tu LC, Naseri A, Huisman M, Zhang S (2016). Multiplexed labeling of genomic loci with dCas9 and engineered sgRNAs using CRISPRainbow. Nat Biotechnol.

[CR9] Qin P, Parlak M, Kuscu C, Bandaria J, Mir M (2017). Live cell imaging of low- and non-repetitive chromosome loci using CRISPR-Cas9. Nat Commun.

[CR10] Schmitt E, Schwarz-Finsterle J, Stein S, Boxler C, Muller P (2010). COMBinatorial Oligo FISH: directed labeling of specific genome domains in differentially fixed cell material and live cells. Methods Mol Biol.

[CR11] Shaner NC, Lambert GG, Chammas A, Ni Y, Cranfill PJ (2013). A bright monomeric green fluorescent protein derived from *Branchiostoma lanceolatum*. Nat Methods.

[CR12] Tanenbaum ME, Gilbert LA, Qi LS, Weissman JS, Vale RD (2014). A protein-tagging system for signal amplification in gene expression and fluorescence imaging. Cell.

[CR13] Wang S, Su JH, Zhang F, Zhuang X (2016). An RNA-aptamer-based two-color CRISPR labeling system. Sci Rep.

[CR14] Wright AV, Nunez JK, Doudna JA (2016). Biology and applications of CRISPR systems: harnessing nature's toolbox for genome engineering. Cell.

