# Supplementary material for: Live cell imaging of genomic loci using dCas9-SunTag system and a bright fluorescent protein
Source: Protein Cell. 2017 Aug 21;8(11):853–5. doi: 10.1007/s13238-017-0460-0 (PMC5676592; doi:10.1007/s13238-017-0460-0)
Supplement: Supplementary file 7 — Supplementary material 7 (PDF 270 kb) [file 13238_2017_460_MOESM7_ESM.pdf]

Supplementary Methods

sgRNA design and cloning

To generate pCAG-scFv-GCN4_V4-sfGFP, the scFv-GCN4_V4-sfGFP sequence was amplified from pHR-scFv-GCN4-sfGFP-GB1-NLS-dWPRE (Addgene Plasmid#60906), and cloned into the pBlueScript vector with a CAG promoter and a bGH poly A sequence (pCAG plasmid) by Gibson Assembly. The forward and reverse primers for PCR reaction are as follows:

5'- ATTTTGGCAAAGAATTTGCTAGCACGCCACCATGGGCCCCGACAT-3'
5'- AGCGAGCTCTAGCCCGGGCGTCGACTTACACCTTGCGCTTCTTCT-3'

To generate pCAG-scFv-GCN4_V4-mNeonGreen, the DNA sequence of mNeonGreen was synthesized from iGene and replaced sfGFP sequence in pCAG-scFv-GCN4_V4-sfGFP. The plasmid pCAG-scFv-GCN4_V4-3XmNeonGreen was constructed by PCR amplifying two fragments of mNeonGreen to be assembled with pCAG-scFv-GCN4_V4-mNeonGreen by Gibson Assembly. The primers used are as follows:

5'-GTACAAGGGTGGAGGTCGGAGCGGCGTGTCCAAGGGCGAAGAGGA-3'
5'-GGACACGCTACCATCGATGCTACCCTTGTACAGCTCGTCCATGCCC-3'
5'-AGGGTAGCATCGATGGTAGCGTGTCCAAGGGCGAAGAGGA-3'
5'-AAGCTTGTACTCTTCACCGGATCCCTTGTACAGCTCGTCCATGCCC-3'

The sequence of 3XmNeonGreen is as follow (linker sequences between mNeonGreens are underlined):

GTGTCCAAGGGCGAAGAGGACAACATGGCCAGCCTGCCTGCCACCCAC
GAGCTGCACATCTTCGGCAGCATCAACGGCGTGGACTTCGACATGGTG
GGACAGGGCACCGGCAACCCCAACGACGGCTACGAGGAACTGAACCTG
AAGTCCACAAAGGGCGACCTGCAGTTCAGCCCCTGGATTCTGGTGCCCC
ACATCGGCTACGGCTTCCACCAGTACCTGCCCTACCCCGACGGCATGAG
CCCTTTCCAGGCCGCTATGGTGGATGGCAGCGGCTACCAGGTGCACCG
GACCATGCAGTTTGAGGACGGCGCCAGCCTGACCGTGAACTACAGATAC
ACCTACGAGGGCAGCCACATCAAGGGCGAGGCCCAAGTGAAGGGCACA
GGCTTTCCAGCCGACGGCCCCGTGATGACCAATAGCCTGACAGCCGCC
GACTGGTGCAGAAGCAAGAAAACCTACCCCAATGACAAGACCATCATCA
GCACCTTCAAGTGGTCCTACACCACCGGCAATGGCAAGCGGTACAGAAG
CACCGCCCGGACCACCTACACCTTCGCCAAACCTATGGCCGCCAACTAC
CTGAAGAACCAGCCTATGTACGTGTTCCGCAAGACCGAGCTGAAGCACT
CCAAGACAGAACTGAACTTCAAAGAGTGGCAGAAAGCCTTCACCGACGT
GATGGGCATGGACGAGCTGTACAAGGGTGGAGGTCGGAGCGGCGTGTC
CAAGGGCGAAGAGGACAACATGGCCAGCCTGCCTGCCACCCACGAGCT
GCACATCTTCGGCAGCATCAACGGCGTGGACTTCGACATGGTGGGACA
GGGCACCGGCAACCCCAACGACGGCTACGAGGAACTGAACCTGAAGTC
CACAAAGGGCGACCTGCAGTTCAGCCCCTGGATTCTGGTGCCCCACATC
GGCTACGGCTTCCACCAGTACCTGCCCTACCCCGACGGCATGAGCCCTT
TCCAGGCCGCTATGGTGGATGGCAGCGGCTACCAGGTGCACCGGACCA

TGCAGTTTGAGGACGGCGCCAGCCTGACCGTGAACTACAGATACACCTA
CGAGGGCAGCCACATCAAGGGCGAGGCCCAAGTGAAGGGCACAGGCTT
TCCAGCCGACGGCCCCGTGATGACCAATAGCCTGACAGCCGCCGACTG
GTGCAGAAGCAAGAAAACCTACCCCAATGACAAGACCATCATCAGCACC
TTCAAGTGGTCCTACACCACCGGCAATGGCAAGCGGTACAGAAGCACCG
CCCGGACCACCTACACCTTCGCCAAACCTATGGCCGCCAACTACCTGAA
GAACCAGCCTATGTACGTGTTCCGCAAGACCGAGCTGAAGCACTCCAAG
ACAGAACTGAACTTCAAAGAGTGGCAGAAAGCCTTCACCGACGTGATGG
GCATGGACGAGCTGTACAAGGGTAGCATCGATGGTAGCGTGTCCAAGG
GCGAAGAGGACAACATGGCCAGCCTGCCTGCCACCCACGAGCTGCACA
TCTTCGGCAGCATCAACGGCGTGGACTTCGACATGGTGGGACAGGGCA
CCGGCAACCCCAACGACGGCTACGAGGAACTGAACCTGAAGTCCACAA
AGGGCGACCTGCAGTTCAGCCCCTGGATTCTGGTGCCCCACATCGGCT
ACGGCTTCCACCAGTACCTGCCCTACCCCGACGGCATGAGCCCTTTCCA
GGCCGCTATGGTGGATGGCAGCGGCTACCAGGTGCACCGGACCATGCA
GTTTGAGGACGGCGCCAGCCTGACCGTGAACTACAGATACACCTACGAG
GGCAGCCACATCAAGGGCGAGGCCCAAGTGAAGGGCACAGGCTTTCCA
GCCGACGGCCCCGTGATGACCAATAGCCTGACAGCCGCCGACTGGTGC
AGAAGCAAGAAAACCTACCCCAATGACAAGACCATCATCAGCACCTTCAA
GTGGTCCTACACCACCGGCAATGGCAAGCGGTACAGAAGCACCGCCCG
GACCACCTACACCTTCGCCAAACCTATGGCCGCCAACTACCTGAAGAAC
CAGCCTATGTACGTGTTCCGCAAGACCGAGCTGAAGCACTCCAAGACAG
AACTGAACTTCAAAGAGTGGCAGAAAGCCTTCACCGACGTGATGGGCAT
GGACGAGCTGTACAAG

To generate pCAG-dCas9-24XGCN4_V4-P2A-BFP, dCas9 and 24XGCN4_V4-P2A-BFP sequences were amplified from pHRdSV40-NLS-dCas9-24xGCN4_v4-NLS-P2A-BFP-dWPRE (Addgene Plasmid#60910) and ligated to pCAG plasmid by Gibson Assembly. The primers for dCas9 amplification are as follows:

5’-GTCTCATCATTTTGGCAAAGGGTACCATGCCCAAGAAGAAG-3’

5’-CGTCGGCCAGGATCACTCTCTTGGAGAATTCGCTGATCTGCTCGATGATC-3’. The primers for 24XGCN4_V4-NLS-P2A-BFP amplification are as follows:

5’ GATCATCGAGCAGATCAGCGAATTCTCCAAGAGAGTGATCCTGGC-3’

5’ ATCCCCGCGCTGCAGTTACTTGTACTTAATTAAGCTTGTGCCCCA-3’

The plasmid pU6-EFsgRNA contains an optimized sgRNA driven by U6 promoter. The optimized sgRNA (F+E) A-U flip & extension of hairpin design is as follow:

GTTT**A**AGAGCTATGCTGGAAACAGCATAGCAAGTTTAAATAAGGCTAGTCCGTTATCAACTTGAAAAAGTGGCACCGAGTCGGTGCT, and A-U flip is in bold and extension of hairpin is underlined (Chen et al., 2013). The CRISPR target sites are listed as follows (PAM sites are bold and underlined):

sgCh5R: GACTGAGGGCCTCCATCGC**GGG**

sgCh14R: GGGACAGGTGGGGACAGCA**TGG**

sgTelomere: GTTAGGGTTAGGGTTAGGGTTA**GGG**

Cell culture and transfection

Human embryonic kidney cell line HEK293T was cultured in Dulbecco's modified Eagle medium(DMEM) with high glucose (Gibco) in 10% (v/v) FBS (Gemcell), 100U/ml penicillin, 100ug/ml streptomycin (Gibco),1XL-Glutamine Solution (Gibco) and 50 μM β-mercaptoethanol and maintained at 37℃ and 5% CO_2 in a humidified incubator. For imaging, cells were grown on 35-mm glass-bottom dishes (MatTek) for 24 hours. Then 1250 ng of dCas9-SunTag24X plasmid, 750 ng indicated sgRNA and 500 ng scFv-GCN4-FP were co-transfected using lipofectamine 3000 (Life Technologies). 48 hours later, cells were analyzed.

Fluorescence in situ hybridization and Immunofluorescence

The labeling protocol was the same as previously described (Schmitt et al., 2010). Briefly, cells were fixed with 4% paraformaldehyde, permeabilized with 0.7% Triton X-100, 0.1% Saponin in 2XSSC, and treated with RNAse A at 37°C for 1h. After dehydration by consecutive 5 min incubations in 70%, 85% and 100% ethanol, cells were heated at 85°C for 30 min in 70% formamide/2XSSC, washed with ethanol series (ice cold; 70, 80, 95%), and labeled with 2 ng/ml Cy5-labeled oligo FISH probe in hybridizing solution (10% dextran sulfate, 50% formamide, 500 ng/ml Salmon sperm DNA in 2XSSC buffer) overnight at 37°C. After washed with 2XSSC for three times, cells were blocked with PB buffer (0.5% BSA, 1XPBS) for 1hr, and consecutively incubated with primary antibody (mouse anti-HA antibody at 1:1000 dilution, MBL: M180-3) overnight at 4°C and with secondary antibody (goat anti-mouse Alexa 488 at 1:2000 dilution, Thermo Fisher A11001) for 1hr at 37°C. Finally, cells were stained with DAPI and analyzed. The sequence of oligo FISH probe for telomere visualization:
Cy5-TTAGGGTTAGGGTTAGGGTTAGGGTTAGGGTTAGGGTTAGGGTTAG
GG-3'

Image acquisition and analysis

All pictures and movies were acquired on a Zeiss LSM880, AxioObserver laser scanning confocal microscope with Plan-Apochromat 100X/1.4 Oil M27 objective. Cells were captured in z-stack with 0.5 μm separation in Fig 1D and 0.4 μm in Fig 1B and 1G. The telomere dynamics were achieved 0.31s per frame. The total number of telomere foci was automatically counted and the telomere trajectory was tracing by Imaris 9.0 (BitPlane) software in each reconstituted 3D cell nucleus. Quantitation of fluorescence intensity was analyzed with ImageJ. ROI, region of interest.

To calculate the fluorescence intensity in Figure1E, the following formula was used:

Fluorescence intensity = Area of ROI X (Mean grey value of ROI – Mean grey value of background)

To calculate the signal-to-noise ratio in Figure1F, the following formula was

used:
    Signal-to-noise = Mean grey value of ROI / Mean grey value of background

Supplementary Figure Legends

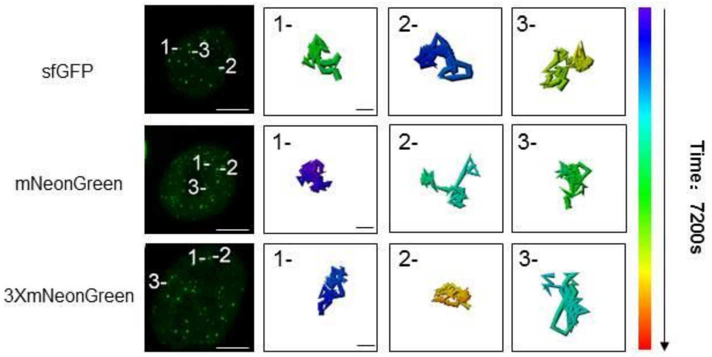

Supplementary Figure

Figure S1. Tracking of telomere dynamics in live cells with CRISPR approaches. Imaging of telomeres in HEK293T cells (Scale bar, 5μm) and trajectories of three representative telomeres (Scale bar, 0.3μm).

Video

Movie S1, S2 and S3. 3D view of telomeres using CRISPR imaging with sfGFP, mNeonGreen and 3XmNeonGreen, respectively.
Movie S4, S5 and S6. Time-series of telomeres using CRISPR imaging with sfGFP, mNeonGreen and 3XmNeonGreen, respectively.
